# Supplementary material for: Modulation of the MOP Receptor (μ Opioid Receptor) by Imidazo[1,2-a]imidazole-5,6-Diones: In Search of the Elucidation of the Mechanism of Action
Source: Molecules. 2022 May 4;27(9):2930. doi: 10.3390/molecules27092930 (PMC9100072; doi:10.3390/molecules27092930)
Supplement: Supplementary file 1 [file molecules-27-02930-s001.zip › molecules-1645771-supplementary.pdf]

Supplementary Materials

Chemical part

1-Phenyl-2,3-dihydro-1*H*-imidazo[1,2-*a*]imidazole-5,6-dione (1a)
Compound **1a** was prepared according to the general procedure using 1-phenyl-2-iminoimidazolidine and diethyl oxolate; a yellow solid with 75% yield was obtained.
Formula: $C_{11}H_9N_3O_2$. (m.m. calc. 215.20). M.p. 278-280 ℃. ^{1}H NMR (δ , ppm, DMSO-d_6, TMS): 3.98 (dd, *J*=7.5 Hz, *J'*= 5 Hz, 2H, C-3), 4.50 (dd, *J*=7.5 Hz, *J'*= 5 Hz, 2H, C-2), 7.30-7.83 (m, 5H, phenyl). ^{13}C NMR (δ, ppm, DMSO-d_6, TMS): 38.38, 51.44, 120.74, 126.50, 129.83, 130.39, 137.06, 158.47, 169.97, 174.59. API-MS: m/z 216,08 ([M+H]$^+$). R_f = 0.37.

1-(3-Methylphenyl)-2,3-dihydro-1*H*-imidazo[1,2-*a*]imidazole-5,6-dione (1b)
Compound **1b** was prepared according to the general procedure using 1-(3-methylphenyl)-2-iminoimidazolidine and diethyl oxolate; a yellow solid with 70% yield was obtained.
Formula: $C_{12}H_{11}N_3O_2$ (m.m. calc. 229.226). M.p. 220-223 ℃. ^{1}H NMR (δ , ppm, DMSO-d_6, TMS): 2.35 (s, 3H, CH_3), 3.95 (dd, *J*=7.5 Hz, *J'*= 5.5 Hz, 2H, C-3), 4.47 (dd, *J*=7.5 Hz, *J'*= 5 Hz, 2H, C-2), 7.11-7.63 (m, 4H , aryl). ^{13}C NMR (δ, ppm, DMSO-d_6, TMS): 21.59, 40.99, 51.49, 117.99, 121.17, 127.19, 129.83, 130.05, 137.03, 139.36, 158.46, 169.93, 174.59. API-MS: m/z 230,09 ([M+H]$^+$). R_f = 0.35.

1-(4-Methylphenyl)-2,3-dihydro-1*H*-imidazo[1,2-*a*]imidazole-5,6-dione (1c)
Compound **1c** was prepared according to the general procedure using 1-(4-methylphenyl)-2-iminoimidazolidine and diethyl oxolate; a yellow solid with 65% yield was obtained.
Formula: $C_{12}H_{11}N_3O_2$ (m.m. calc. 229.226). M.p. 236-238 ℃. ^{1}H NMR (δ, ppm, DMSO-d_6, TMS): 2.32 (s, 3H, CH_3), 3.94 (dd, *J*=7.5 Hz, *J'*= 5 Hz, 2H, C-3), 4.46 (dd, *J*=7.5 Hz, *J'*= 5 Hz, 2H, C-2), 7.30-7.70 (2 x d, *J*=8 Hz 4H, aryl). ^{13}C NMR (δ, ppm, DMSO-d_6, TMS): 20.92, 37.78, 51.50, 120.62, 130.01, 134.59, 135.97, 158.74, 169.54, 174.59. API-MS: m/z 230,09 ([M+H]$^+$). R_f = 0.34.

1-(3-Chlorophenyl)-2,3-dihydro-1*H*-imidazo[1,2-*a*]imidazole-5,6-dione (1d)
Compound **1d** was prepared according to the general procedure using 1-(3-chlorophenyl)-2-iminoimidazolidine and diethyl oxolate; a yellow solid with 58% yield was obtained.
Formula: $C_{11}H_8ClN_3O_2$ (m.m. calc. 249.645). M.p. 279-280 ℃. ^{1}H NMR (δ, ppm, DMSO-d_6, TMS): 3.98 (dd, *J*=7.5 Hz, *J'*= 5 Hz, 2H, C-3), 4.47 (dd, *J*=7.5 Hz, *J'*= 5 Hz, 2H, C-2), 7.30-7.65 (m, 4H, aryl). ^{13}C NMR (δ, ppm, DMSO-d_6, TMS): 38.44, 51.32, 122.33, 127,59, 130.31, 136.05, 158.39, 170.20, 174.41. API-MS: m/z 250,04 ([M+H]$^+$). R_f = 0.32.

1-(4-chlorophenyl)-2,3-dihydro-1*H*-imidazo[1,2-*a*]imidazole-5,6-dione (1e)
Compound **1e** was prepared according to the general procedure using 1-(4-chlorophenyl)-2-iminoimidazolidine and diethyl oxolate; a yellow solid with 52% yield was obtained.
Formula: $C_{11}H_8ClN_3O_2$ (m.m. calc. 249.645). M.p. 260-263 ℃. ^{1}H NMR (δ, ppm, DMSO-d_6, TMS): 4.05 (dd, *J*=7.5 Hz, *J'*= 5 Hz, 2H, C-3), 4.45 (dd, *J*=7.5 Hz, *J'*= 5 Hz, 2H, C-2), 7.25-7.72 (2 x d, *J*=9 Hz, 4H, aryl). ^{13}C NMR (δ, ppm, DMSO-d_6, TMS): 38.12, 51.48, 117.92, 129,64, 137.03, 139.365, 158.78, 169.85, 174.34. API-MS: m/z 250,04 ([M+H]$^+$). R_f = 0.31.

1-(2-methoxyphenyl)-2,3-dihydro-1*H*-imidazo[1,2-*a*]imidazole-5,6-dione (1f)
Compound **1f** was prepared according to the general procedure using 1-(2-methoxyphenyl)-2-iminoimidazolidine and diethyl oxolate; a yellow solid with 48% yield was obtained.
Formula: $C_{12}H_{11}N_3O_3$ (m.m. calc. 245.226). M.p. 245-248 ℃. ^{1}H NMR (δ, ppm, DMSO-d_6, TMS): 3.8 (s, 3H, OCH_3), 3.98 (dd, *J*=7.5 Hz, *J'*= 5 Hz, 2H, C-3), 4.39 (dd, *J*=7.5 Hz, *J'*= 5 Hz, 2H, C-2), 7.04-7.48 (m, 4H, aryl). ^{13}C NMR (δ, ppm, DMSO-d_6, TMS): 51.42, 56.47, 113.28, 121.14, 123,73, 128.61, 130.80, 154.72, 159.26, 171.75, 174.78. API-MS: m/z 246,09 ([M+H]$^+$). R_f = 0.40.

1-(4-methoxyphenyl)-2,3-dihydro-1*H*-imidazo[1,2-*a*]imidazole-5,6-dione (1g)
Compound **1g** was prepared according to the general procedure using 1-(4-methoxyphenyl)-2-iminoimidazolidine and diethyl oxolate; a yellow solid with 42% yield was obtained.
Formula: $C_{12}H_{11}N_3O_3$ (m.m. calc. 245.226). M.p. 233-235 ℃. ^{1}H NMR (δ, ppm, DMSO-d_6, TMS): 3.2 (s, 3H, OCH_3), 4.0 (dd, *J*=7.5 Hz, *J'*= 5 Hz, 2H, C-3), 4.35 (dd, *J*=7.5 Hz, *J'*= 5 Hz, 2H, C-2), 7.32-7.65 (2 x d, *J*=9 Hz, 4H, aryl). ^{13}C NMR (δ, ppm, DMSO-d_6, TMS): 38.34 51.48, 56.46, 117.93, 121.10, 127,18, 129.64, 137.03, 158.46, 169.85, 174.58. API-MS: m/z 246.09 ([M+H]$^+$). R_f = 0.39.

Compound 1a
^{1}H NMR spectrum

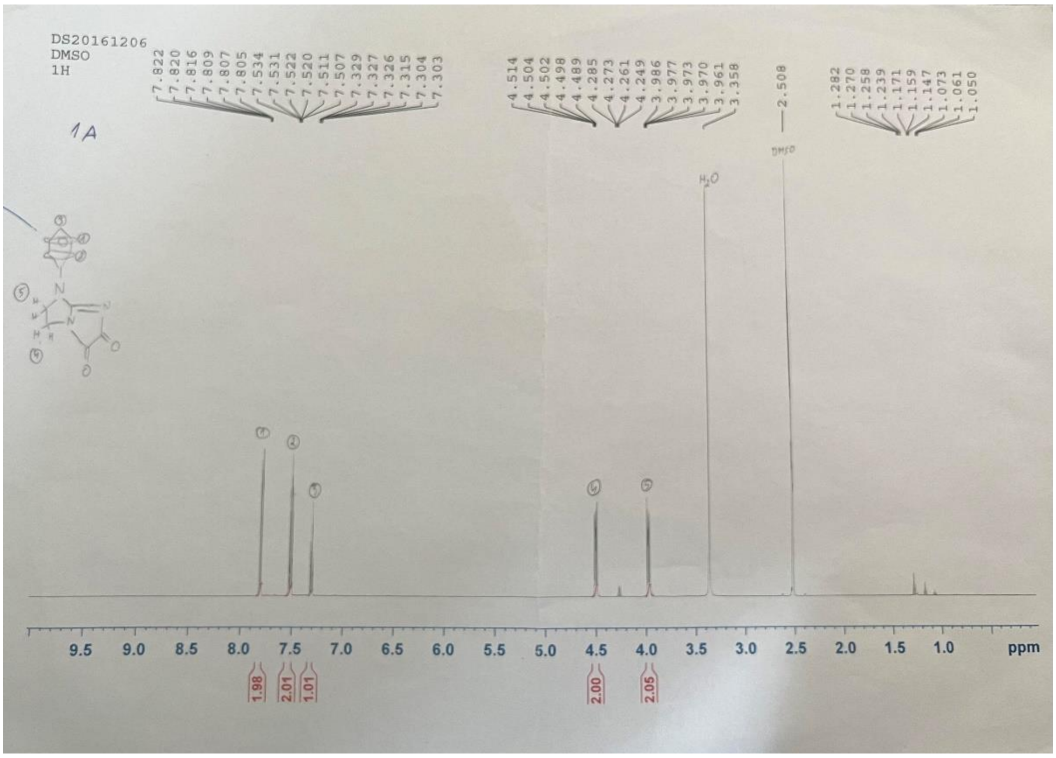

^{13}C NMR spectrum

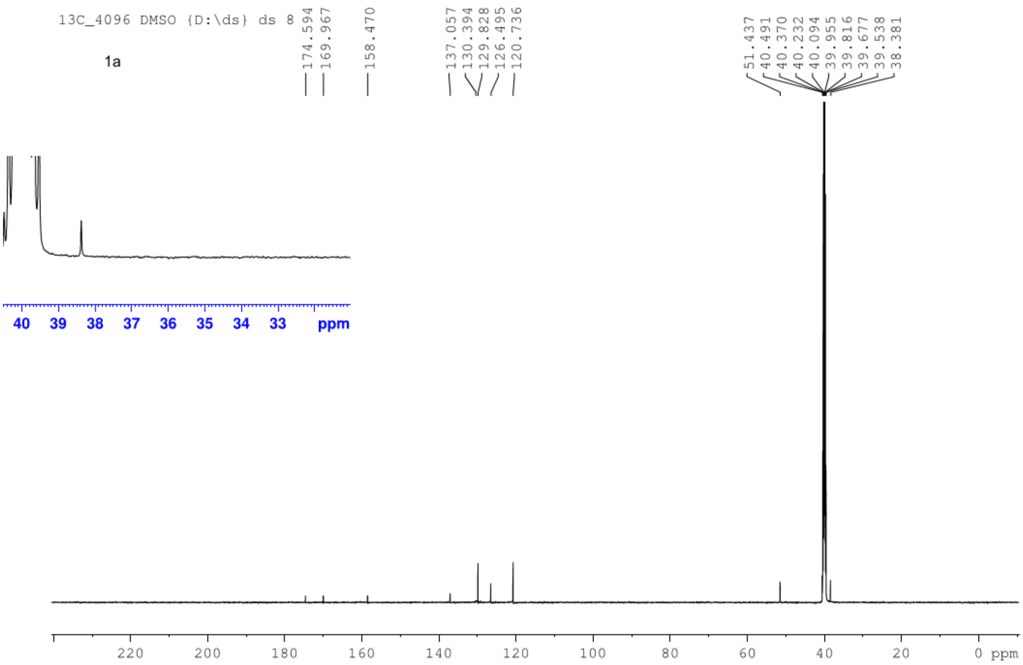

MS spectrum

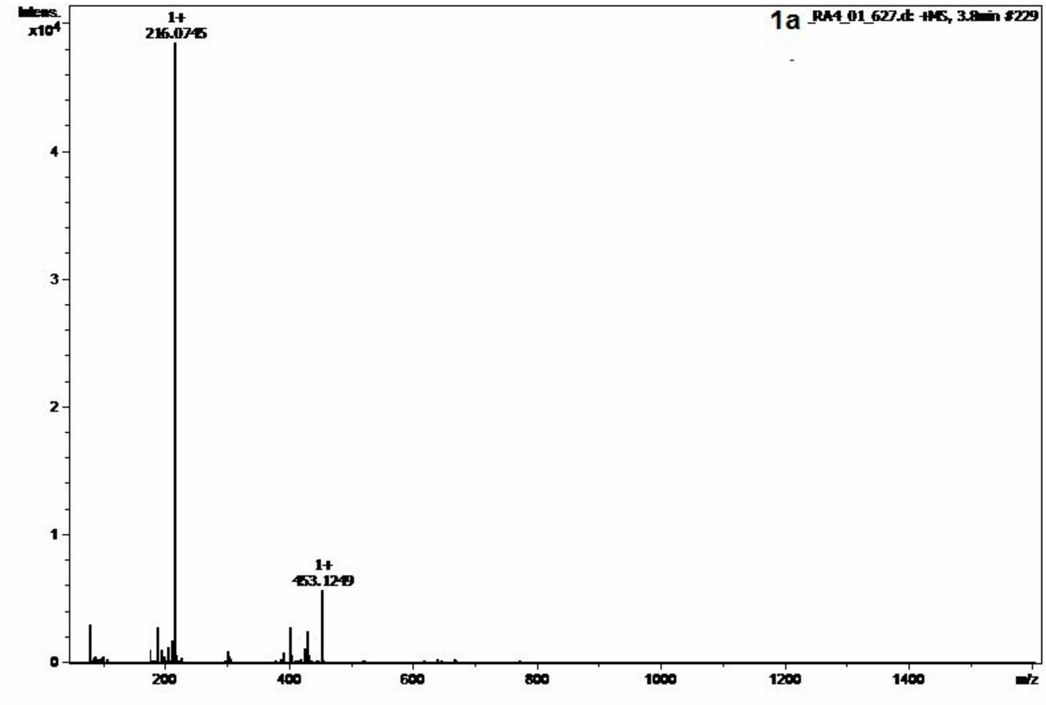

Compound 1b

^{1}H NMR spectrum

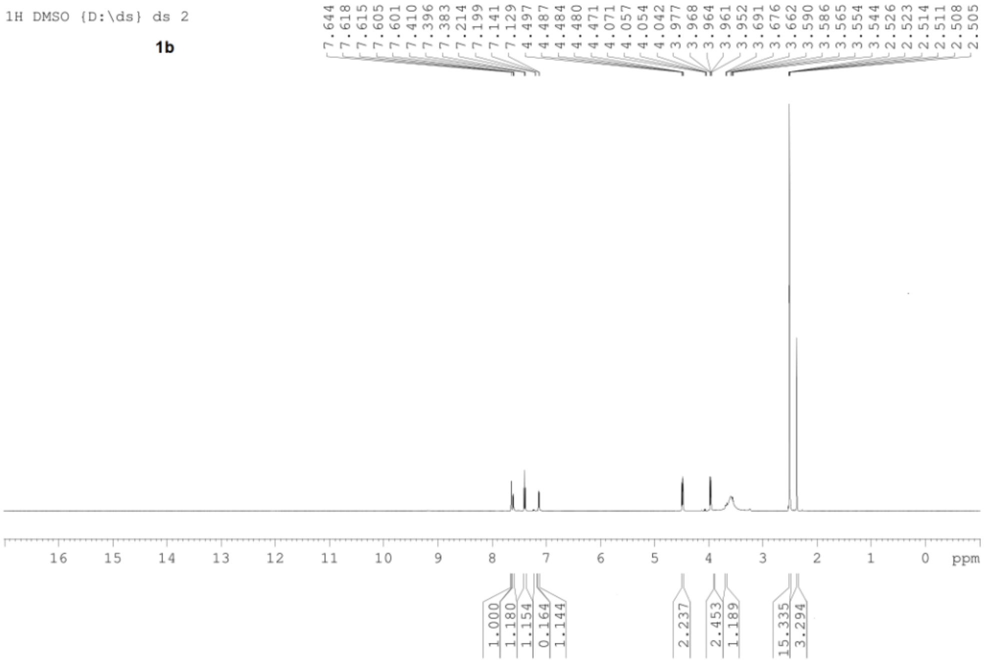

^{13}C NMR spectrum

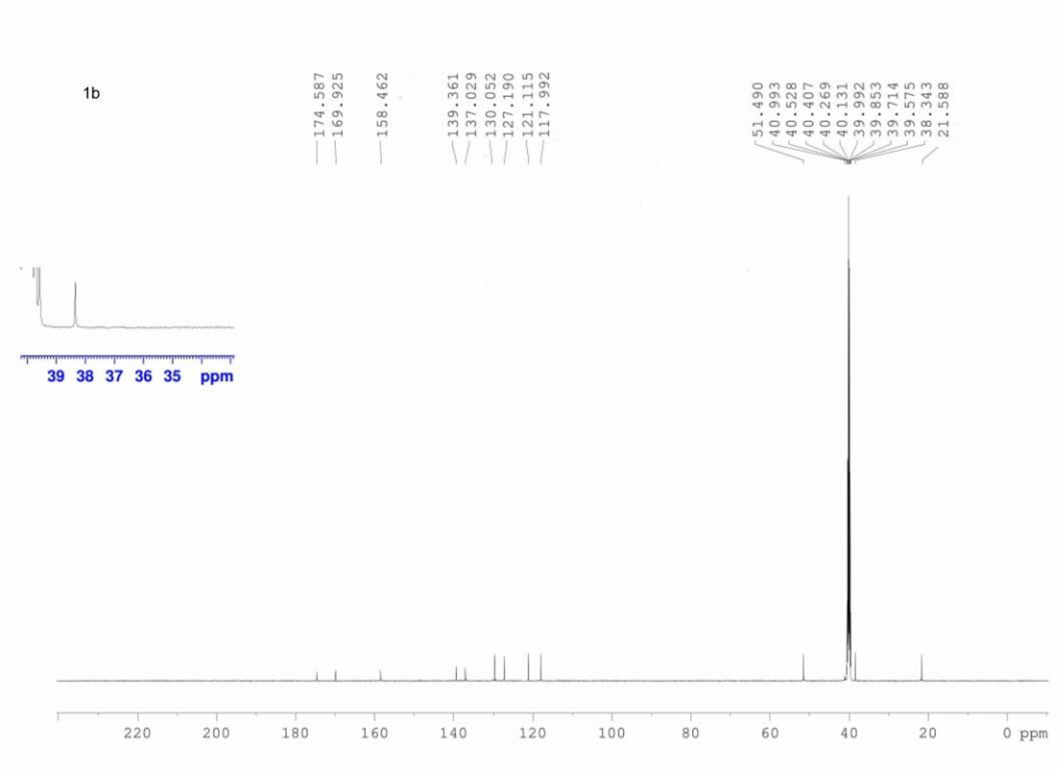

MS spectrum

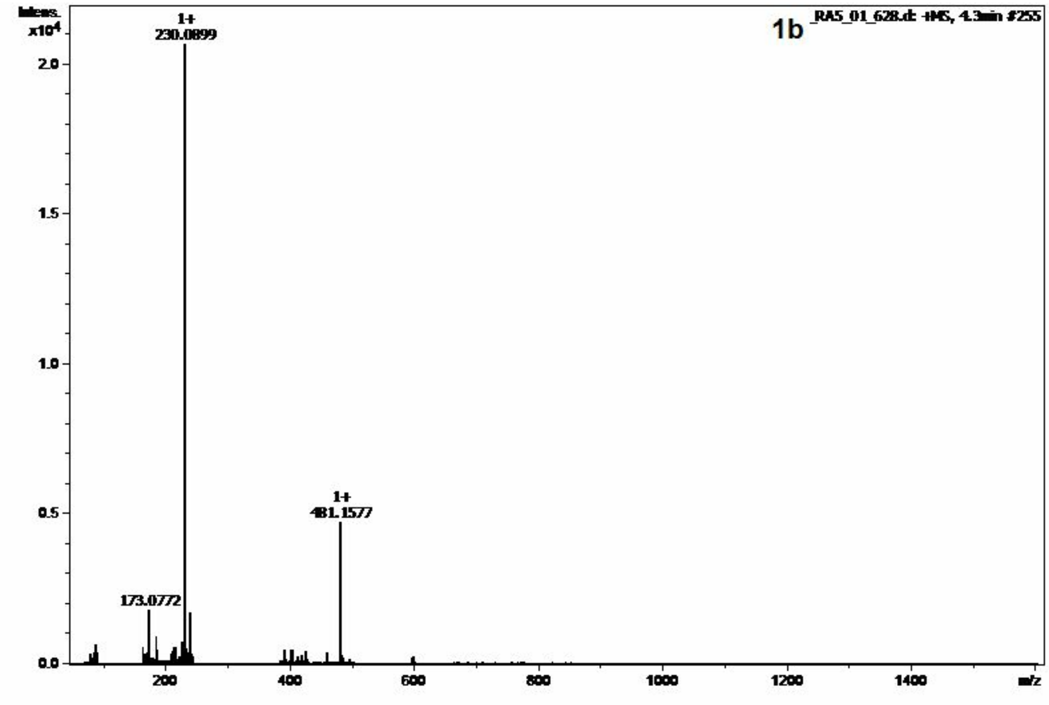

Compound 1c

^{1}H NMR spectrum

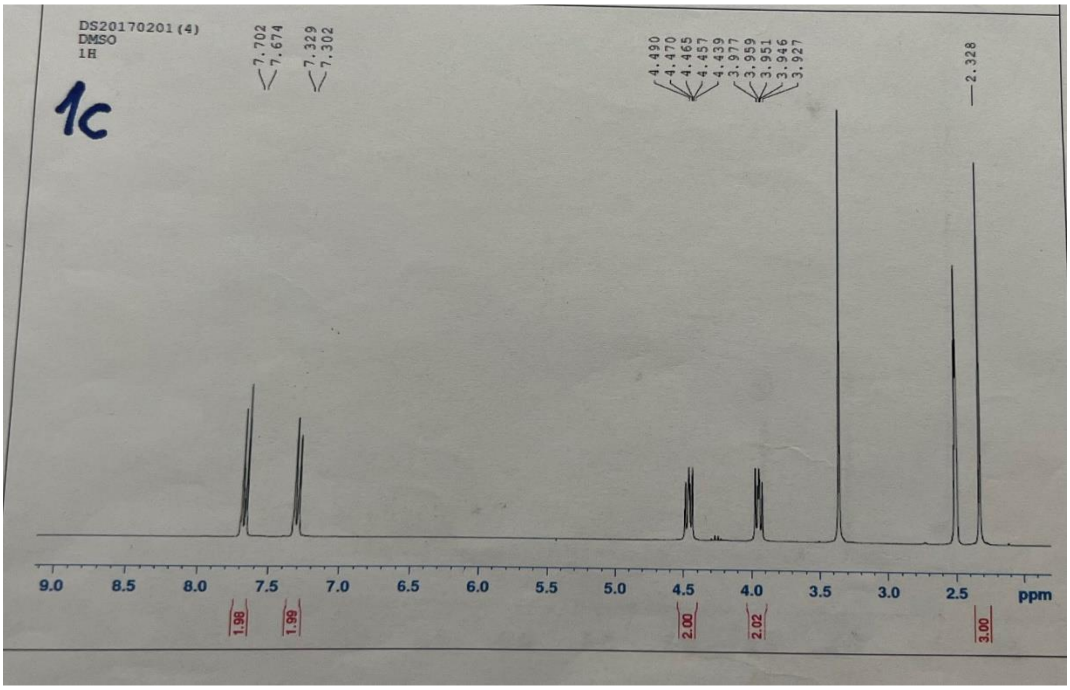

^{13}C NMR spectrum

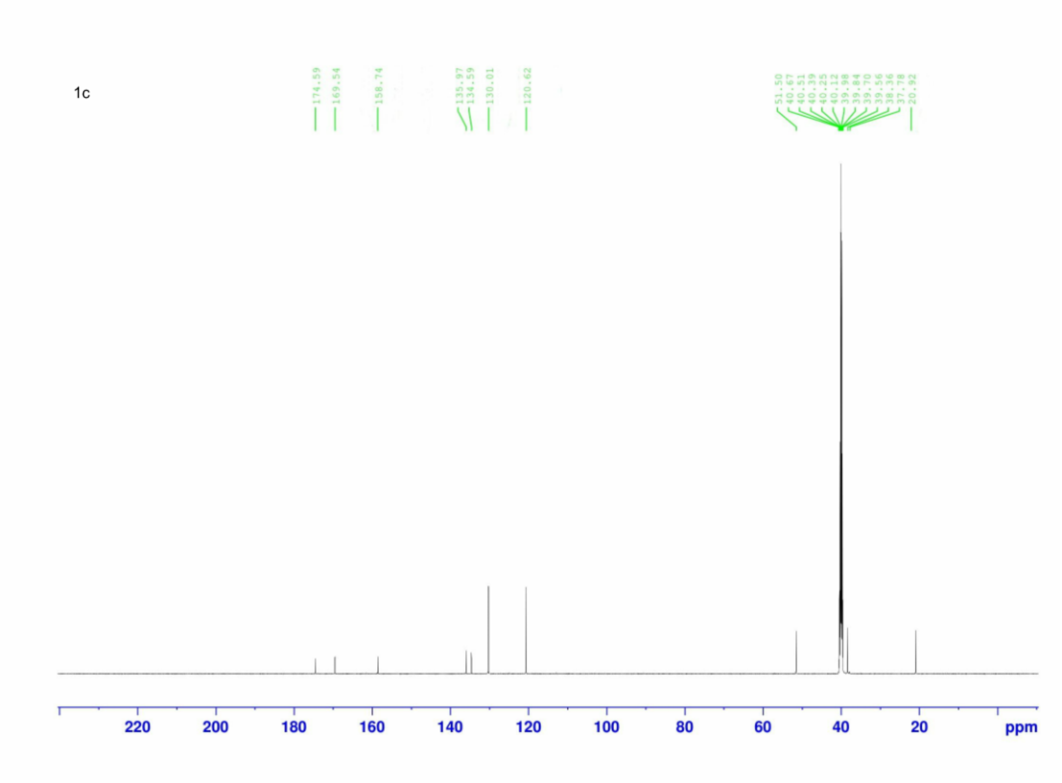

MS spectrum

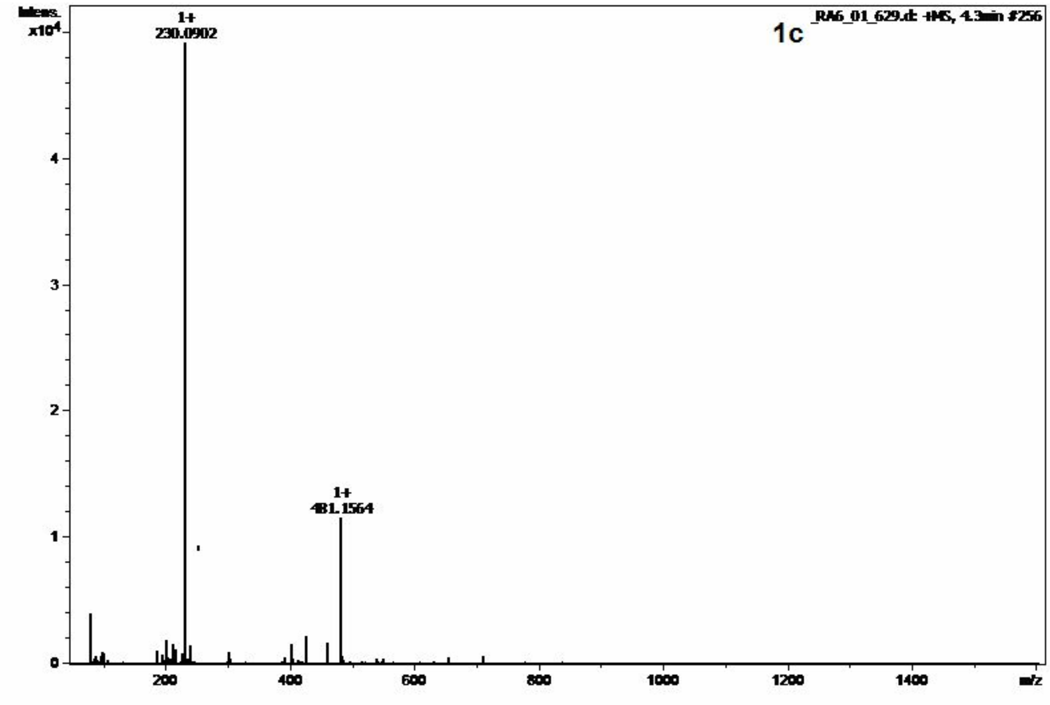

Compound 1d

^{1}H NMR spectrum

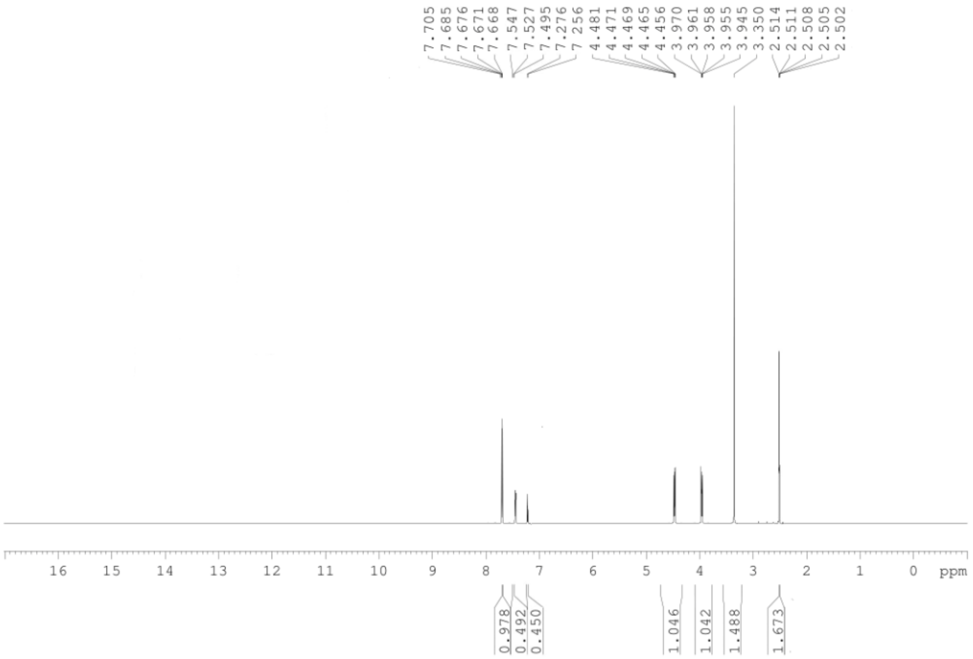

^{13}C NMR spectrum

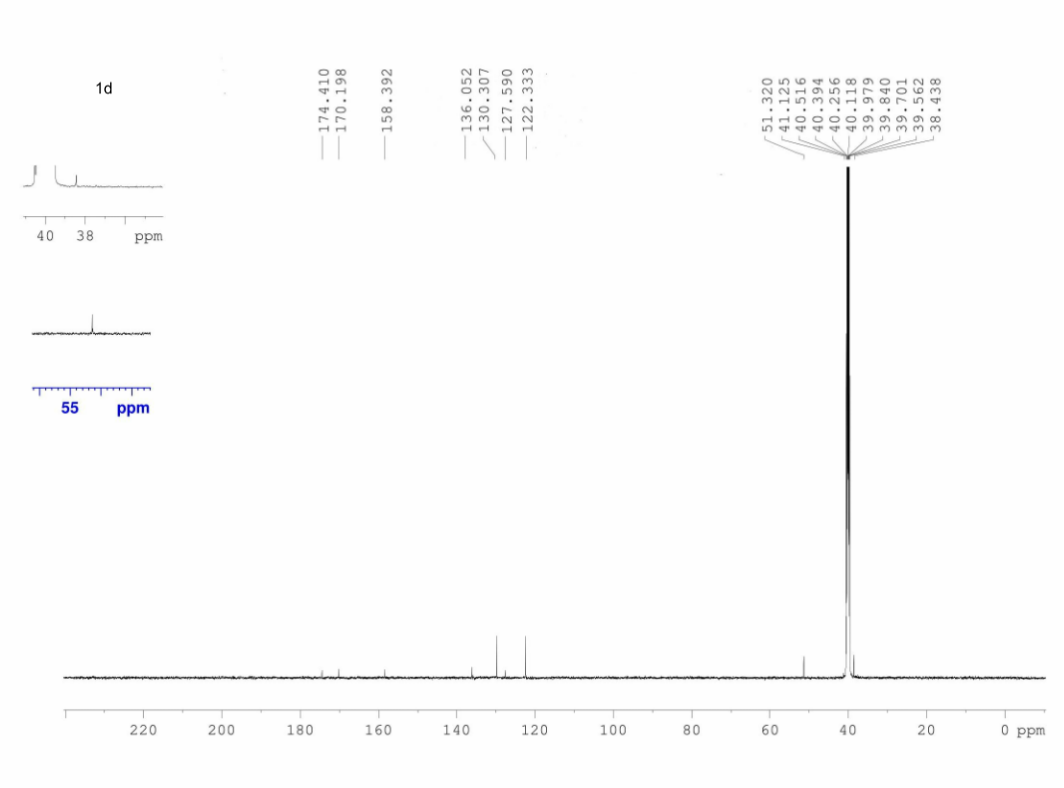

MS spectrum

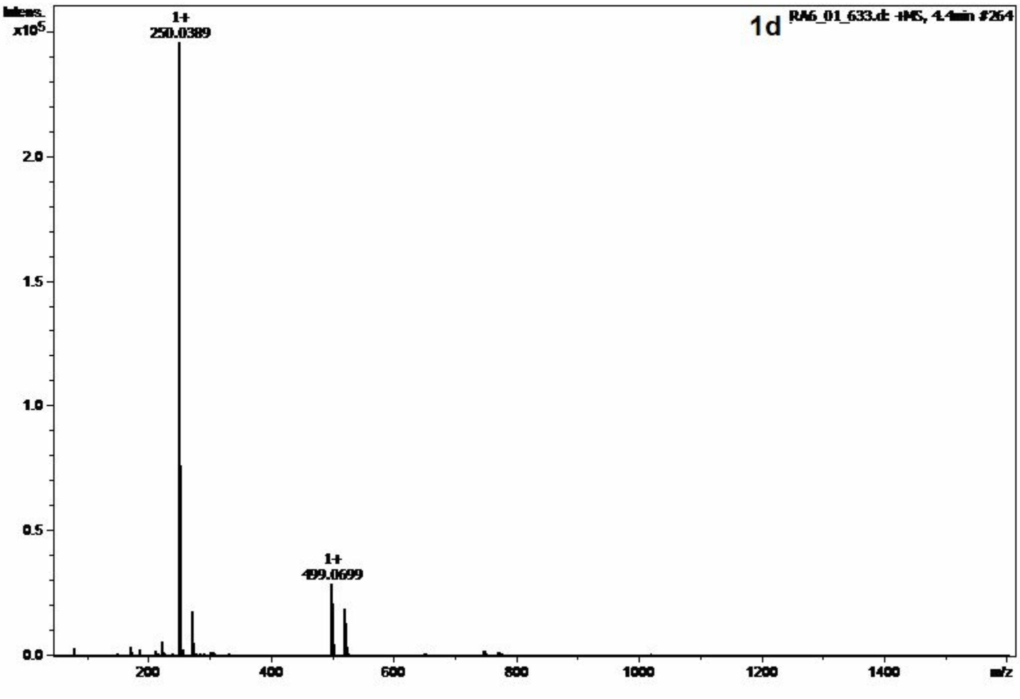

Compound 1e

^{1}H NMR spectrum

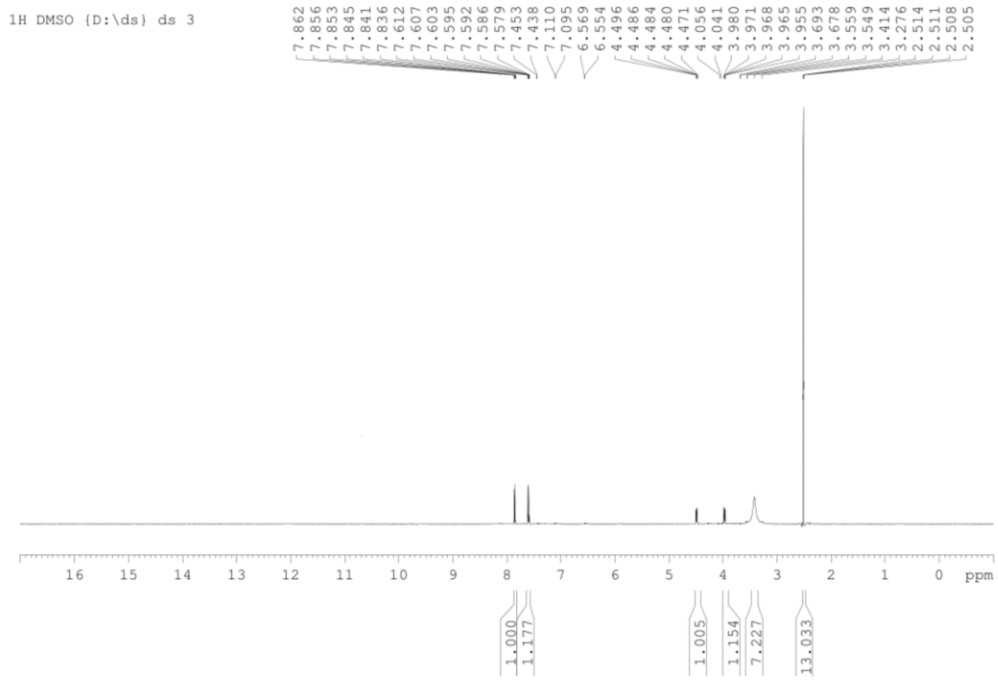

^{13}C NMR spectrum

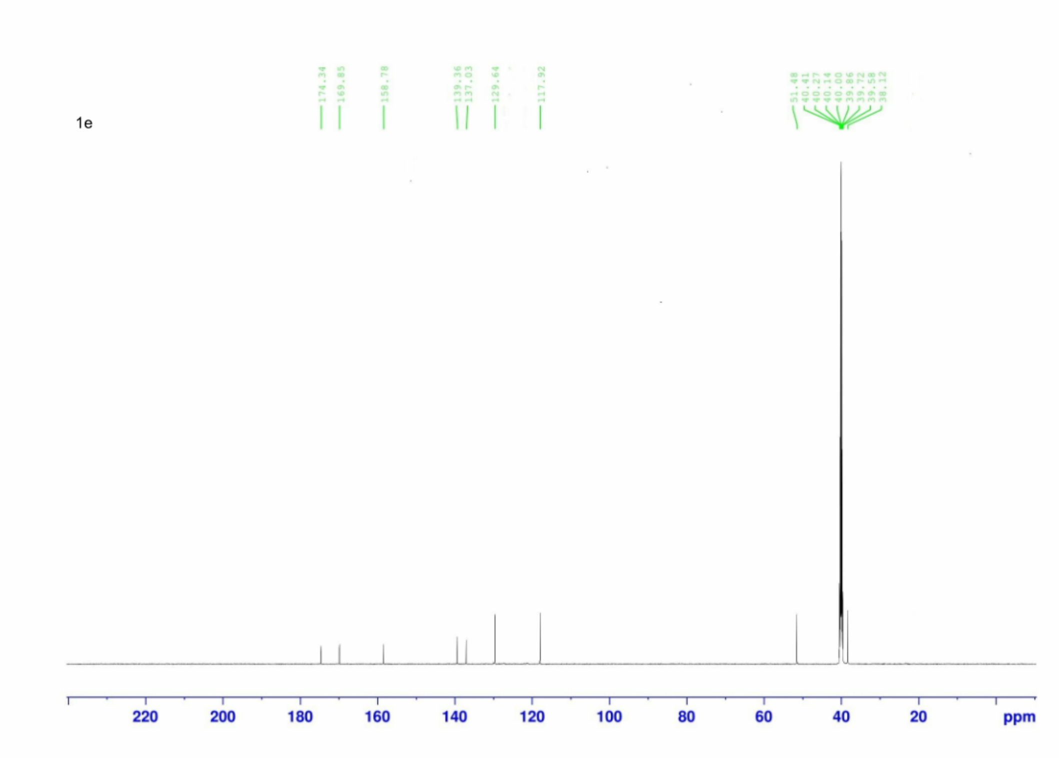

MS spectrum

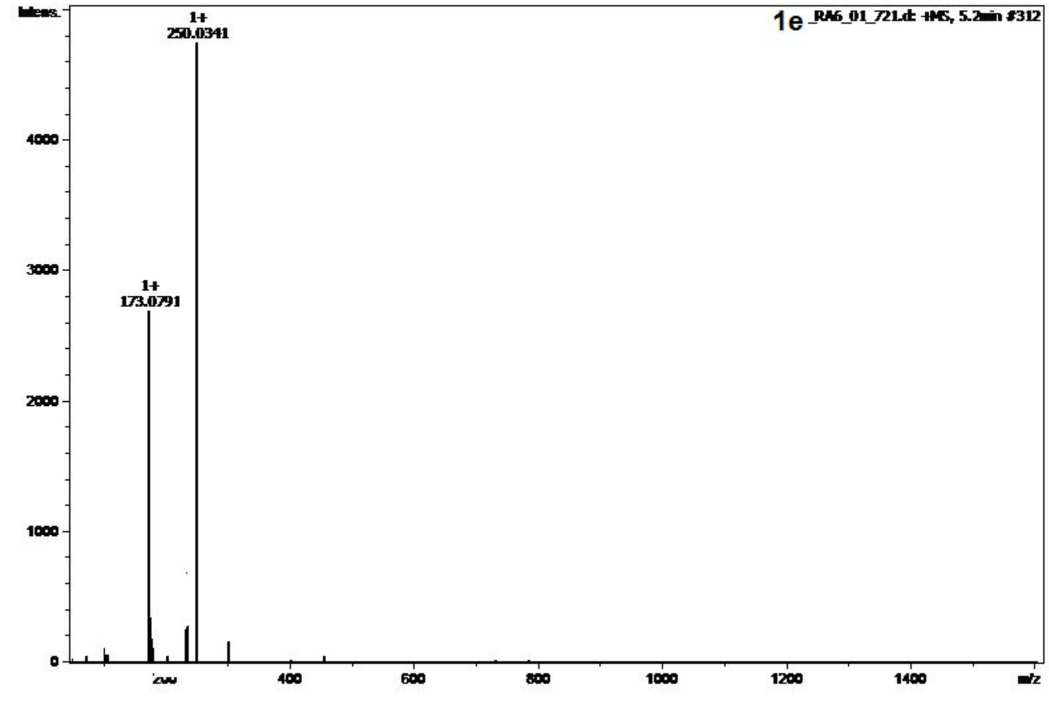

Compound 1f

^{1}H NMR spectrum

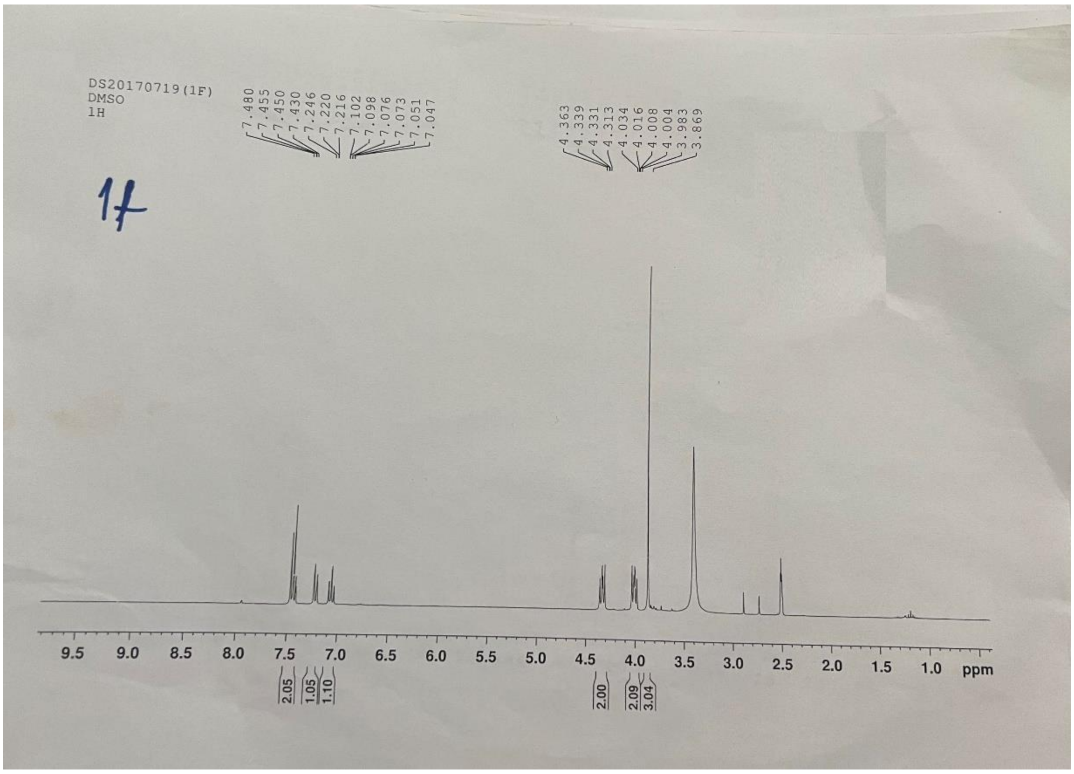

^{13}C NMR spectrum

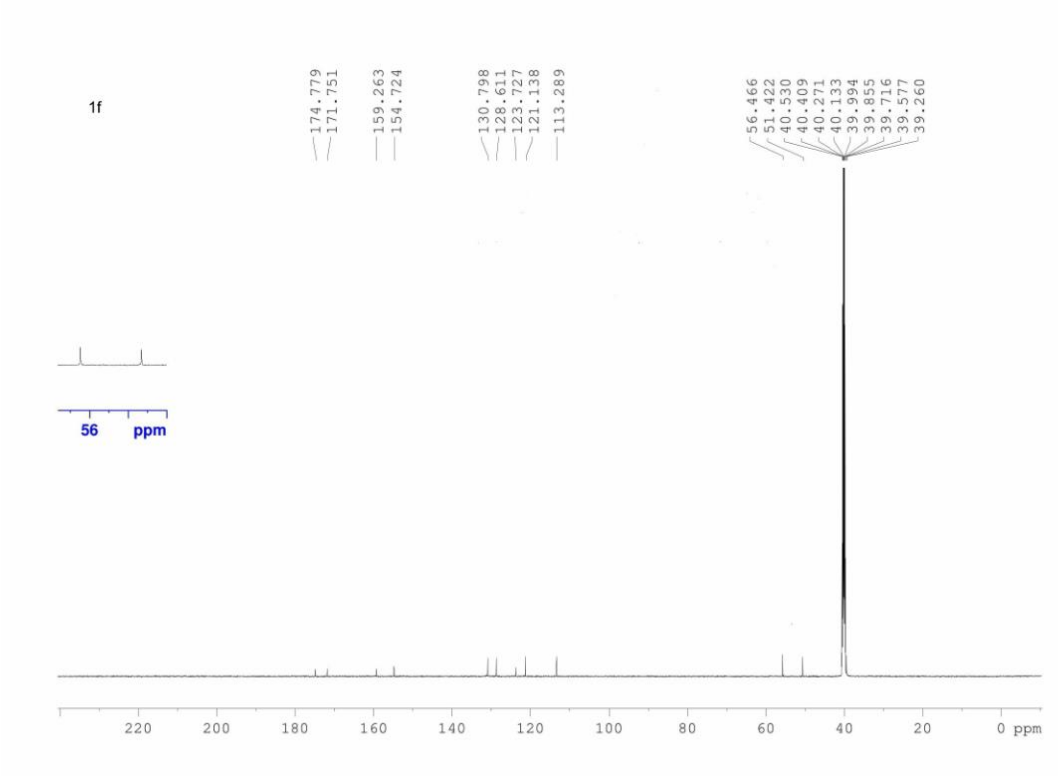

MS spectrum

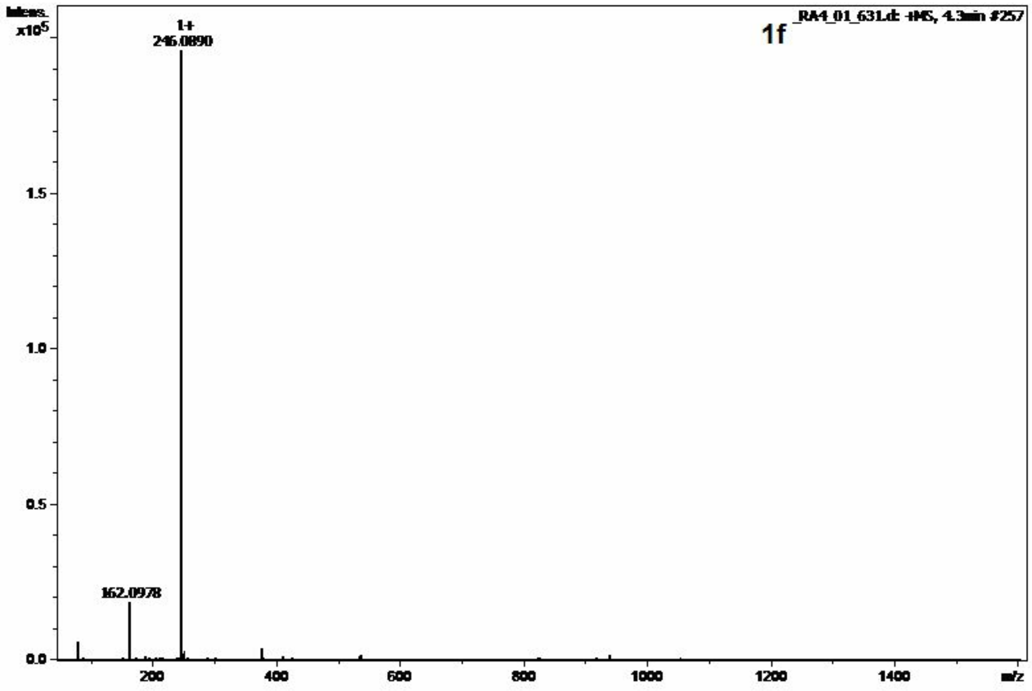

Compound 1g

^{1}H NMR spectrum

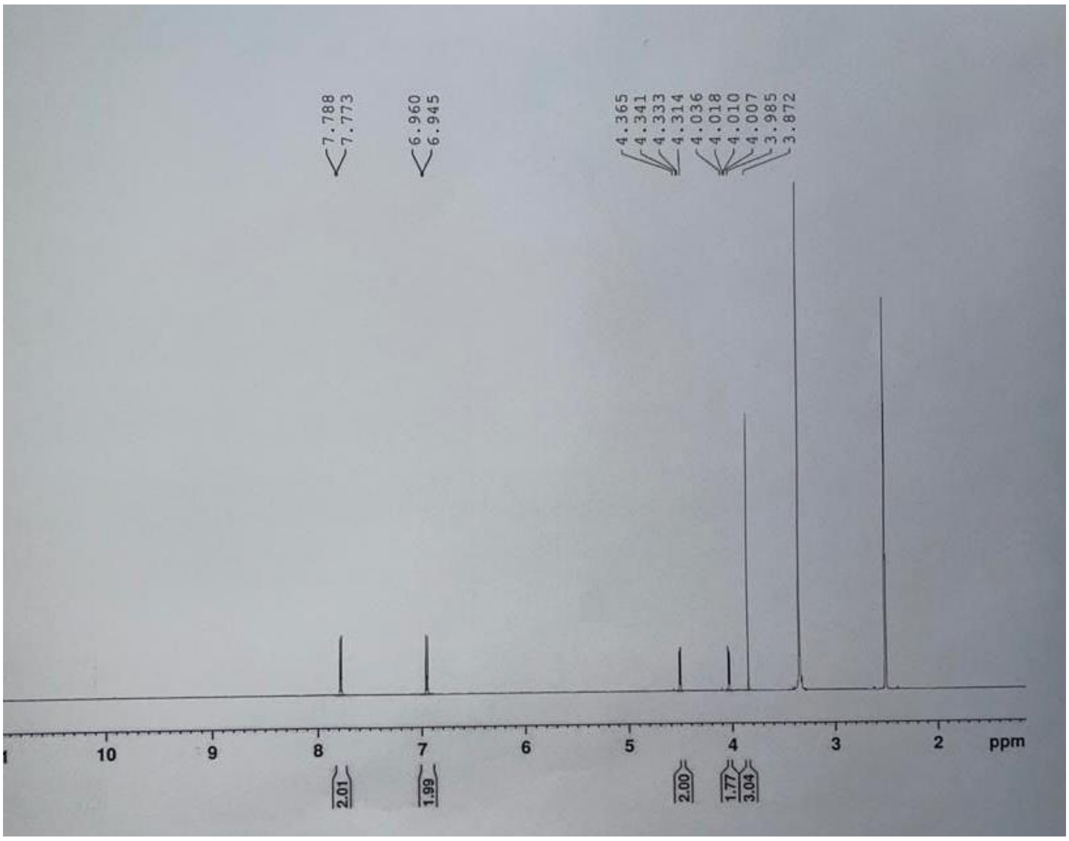

^{13}C NMR spectrum

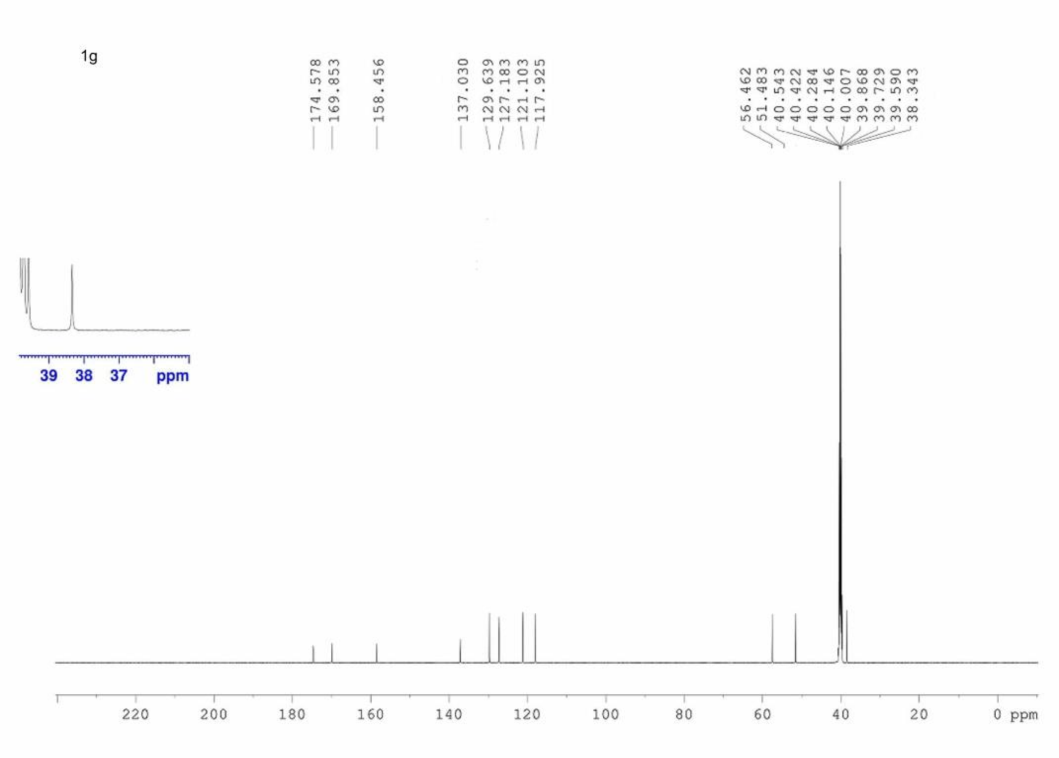

MS spectrum

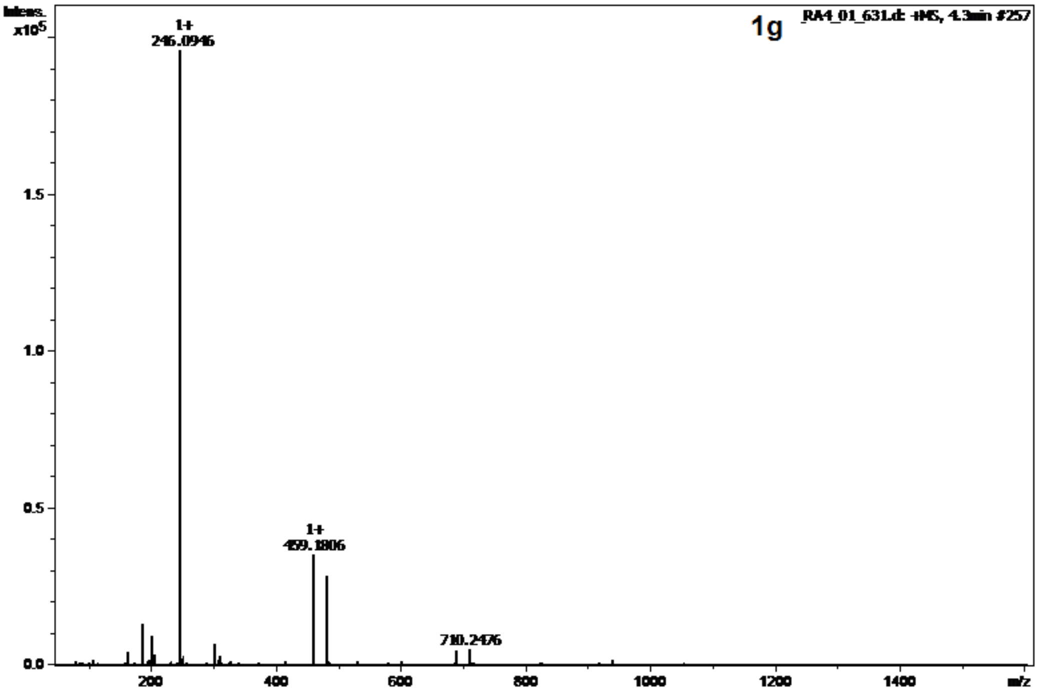

Intens.
x10^5
1g
RA4_01_631.d: +MS, 4.3min #257
1+
246.0946
1+
459.1806
710.2476
1.5
1.0
0.5
0.0
200
400
600
800
1000
1200
1400
m/z

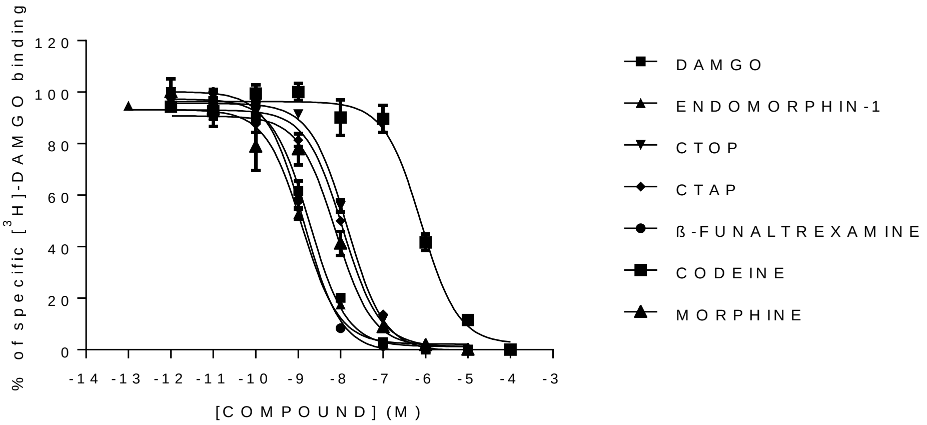

Figure S1. Results of binding of reference compounds to MOP

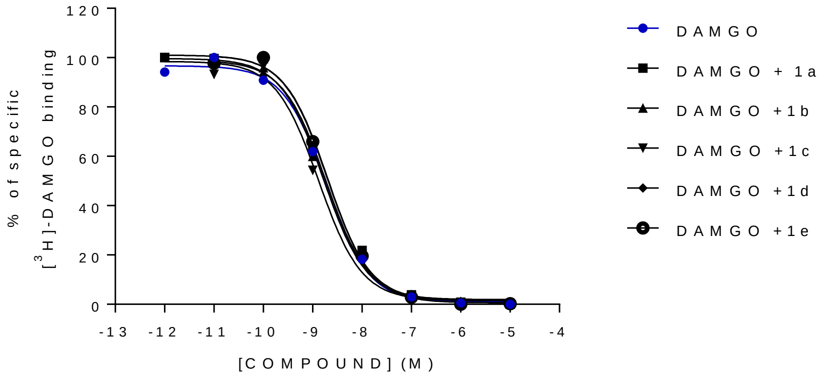

Figure S2. Effect of the tested compounds on the affinity of DAMGO for the MOP. Membranes were incubated with DAMGO at different concentrations (10^{-5}-10^{-12}) in the presence of 0.5 nM of [^{3}H]-DAMGO and the tested compounds at a concentration of 10^{-6} M.

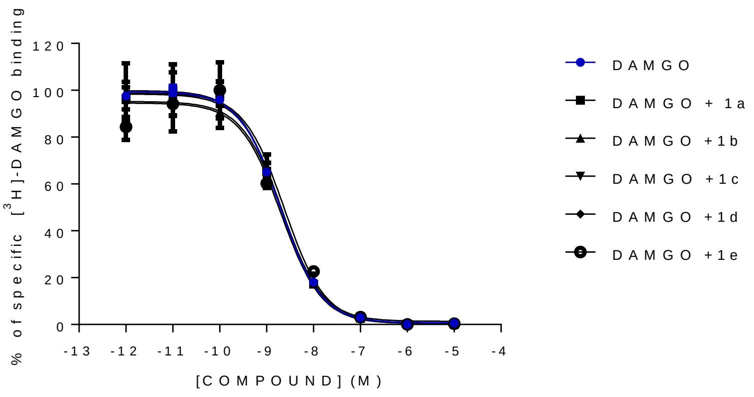

Figure S3. Effect of the tested compounds on the affinity of DAMGO for the MOP. Membranes were incubated with DAMGO at different concentrations (10^{-5}-10^{-12}) in the presence of 0.5 nM of [^{3}H]-DAMGO and the tested compounds at a concentration of 10^{-7} M.

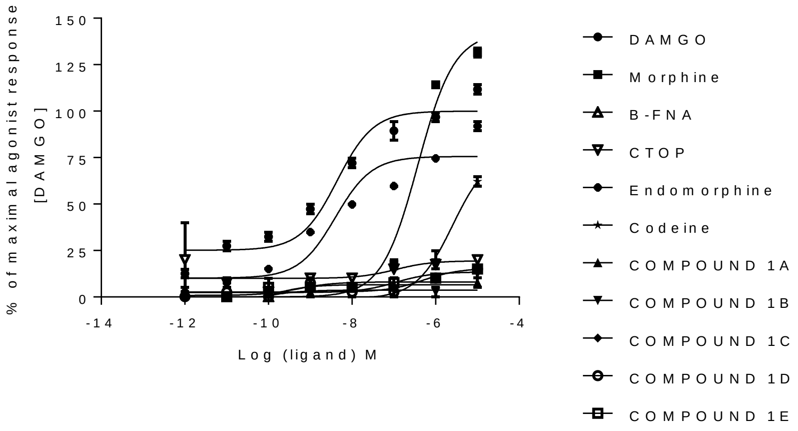

Figure S4. Effect of the tested compounds on the recruitment of β-arrestin in the agonist mode in U2OS cells with stable expression of the MOP

A

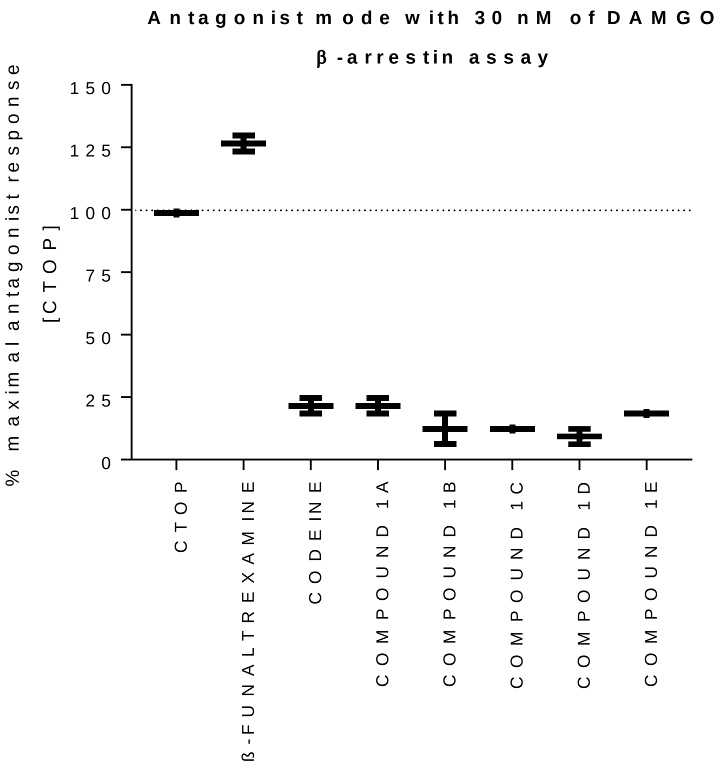

B

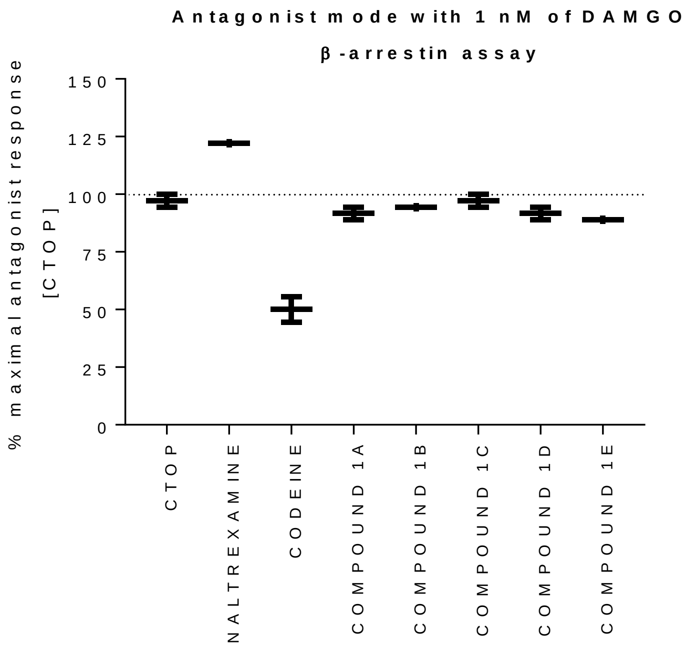

Figure S5. Effect of 10 μM of the tested compounds on the level of β-arrestin in U2OS cells with stable expression

of the MOP; antagonist mode with EC_{87}= 30 nM (A) and EC_{20}= 1 nM (B) concentration of DAMGO.

Spectrum absorbance

A)

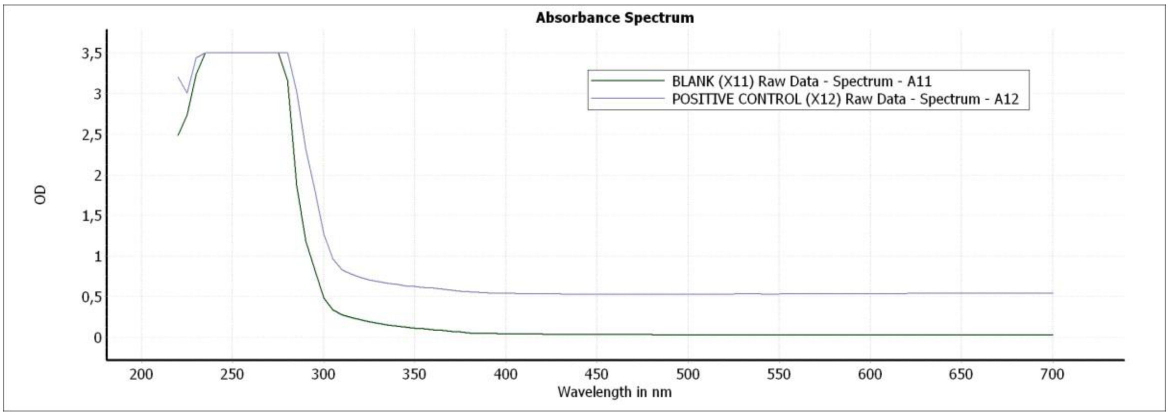

B)

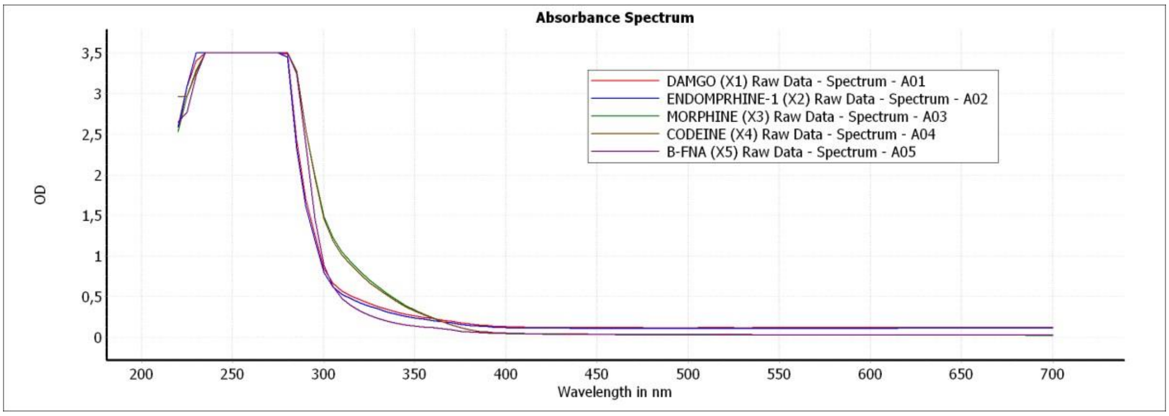

C)

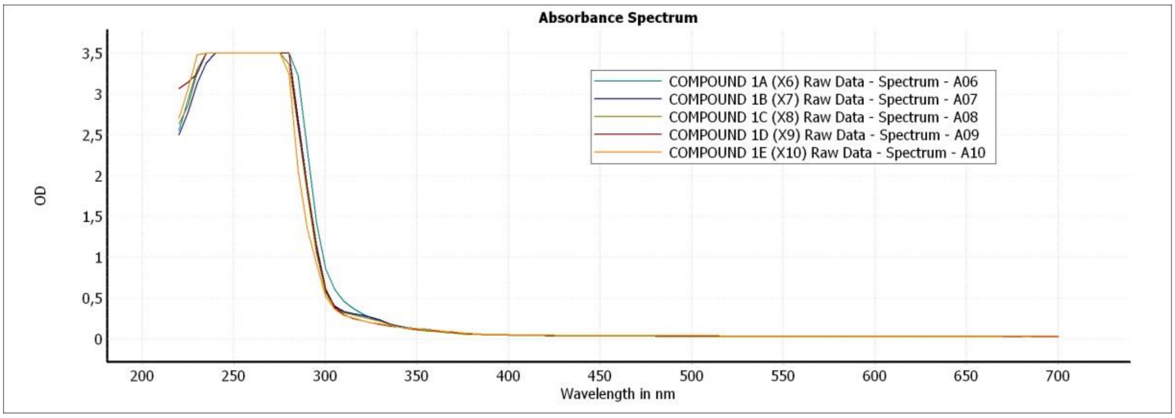

Figure S6. Absorbance spectrum of the controls (A), references (B), and compounds (C) in the range of 220 nm to 700 nm. The spectrum was cut at the 3.5 OD scale due to the sharpness of the absorption signal which would obscure the spectral differences between the reference and tested compounds.
